# The Use of Continuous Glucose Monitoring Combined with Computer-Based eMPC Algorithm for Tight Glucose Control in Cardiosurgical ICU 

**DOI:** 10.1155/2013/186439

**Published:** 2013-02-20

**Authors:** Petr Kopecký, Miloš Mráz, Jan Bláha, Jaroslav Lindner, Štĕpán Svačina, Roman Hovorka, Martin Haluzík

**Affiliations:** ^1^Department of Anaesthesia, Resuscitation and Intensive Medicine, 1st Faculty of Medicine and General University Hospital, Charles University in Prague, U Nemocnice 2, 128 08 Prague 2, Czech Republic; ^2^Third Department of Medicine, 1st Faculty of Medicine and General University Hospital, Charles University in Prague, U Nemocnice 1, 128 08 Prague 2, Czech Republic; ^3^Department of Cardiac Surgery, 1st Faculty of Medicine and General University Hospital, Charles University in Prague, U Nemocnice 2, 128 08 Prague 2, Czech Republic; ^4^Institute of Metabolic Science, University of Cambridge, Addenbrooke's Hospital, Box 289, Cambridge CB2 0QQ, UK

## Abstract

*Aim*. In postcardiac surgery patients, we assessed the performance of a system for intensive intravenous insulin therapy using continuous glucose monitoring (CGM) and enhanced model predictive control (eMPC) algorithm. *Methods*. Glucose control in eMPC-CGM group (*n* = 12) was compared with a control (C) group (*n* = 12) treated by intravenous insulin infusion adjusted according to eMPC protocol with a variable sampling interval alone. In the eMPC-CGM group glucose measured with a REAL-Time CGM system (Guardian RT) served as input for the eMPC adjusting insulin infusion every 15 minutes. The accuracy of CGM was evaluated hourly using reference arterial glucose and Clarke error-grid analysis (C-EGA). Target glucose range was 4.4–6.1 mmol/L. *Results*. Of the 277 paired CGM-reference glycemic values, 270 (97.5%) were in clinically acceptable zones of C-EGA and only 7 (2.5%) were in unacceptable D zone. Glucose control in eMPC-CGM group was comparable to C group in all measured values (average glycemia, percentage of time above, within, and below target range,). No episode of hypoglycemia (<2.9 mmol) occurred in eMPC-CGM group compared to 2 in C group. *Conclusion*. Our data show that the combination of eMPC algorithm with CGM is reliable and accurate enough to test this approach in a larger study population.

## 1. Introduction

Stress hyperglycemia (e.g., “diabetes of injury”) is a common finding in critical care occurring in up to 90% of patients with critical illness [1, 2]. It is associated with increased morbidity and mortality and poorer prognosis of these patients [1–3]. In 2001, the landmark Leuven study performed in cardiosurgical intensive care unit (ICU) demonstrated that intensive insulin therapy (IIT) aimed at maintaining glycemia between 4.4 and 6.1 mmol/L reduced mortality and decreased frequency of severe organ complications [3]. Several other studies confirmed these findings especially in cardiac surgery patients [4]. However, some of the more recent trials questioned safety, reproducibility, and universality of beneficial effects of tight glycemic control (TGC) in other subgroups of critically ill patients [5–7], leading consequently to a shift towards a less intensive approach to glucose lowering in ICU settings in the last years.

Principally, the need to decrease pathologically elevated glycemia in critically ill subjects has been generally accepted, although the exact target range in various patient subgroups is subject of ongoing discussion [1]. Numerous protocols for IIT of variable effectiveness have been developed [1], with most-recently introduced computer-based predictive algorithms showing significantly better performance with less adverse effects compared to standard paper-based protocols [8, 9]. The main complication limiting the use of TGC procedures is the occurrence of hypoglycemia, which was associated with increased risk of death and prolonged ICU stay in several studies [5–7]. As the response of ICU patients to hypoglycemia is often blunted [1], frequent glucose monitoring is an essential prerequisite of nearly all IIT algorithms. However, frequent blood sampling increases dramatically the workload of the nursing staff and the intermittent fashion, in which glucose values are obtained, may not always capture significant hypoglycemic events. Continuous glucose monitoring (CGM) could therefore be an ideal tool for catching rapid glycemic excursions (both hypo- and hyperglycemia) and providing the algorithm with (nearly) real-time glycemic data in order to improve its efficacy and safety. 

To our knowledge only a minimum number of studies including CGM as input for TGC in the ICU and utilizing predominantly nonpredictive IIT protocols have been performed so far [10–13]. To this end, we performed a single-center randomized open-label trial using a combination of the established computer-based eMPC (enhanced model predictive control) algorithm with a standard system for continuous glucose monitoring Guardian REAL-Time CGMS (MiniMed Medtronic, Northridge, CA, USA). We evaluated the efficacy and safety of the combined system and compared it with the performance of the eMPC algorithm alone.

## 2. Research Design and Methods

### 2.1. Study Design and Subjects

The study was designed as a single-center open-label randomized trial. 24 adult patients (16 men and 6 women, aged 46 to 83 years, 5 patients with type 2 diabetes mellitus) undergoing major elective cardiac surgery (aortocoronary bypass or valvular plastic) were enrolled into the study. Twelve patients were randomized for intensive insulin treatment with the eMPC (enhanced model predictive control) protocol combined with continuous glucose measurement (eMPC-CGM group) and 12 were randomized for insulin treatment according to eMPC algorithm alone, which was routinely used at the Department of Cardiovascular Surgery, General University Hospital, Prague. Exclusion criteria were defined as follows: insulin allergy and inability to sign informed consent. Characteristics of both groups are shown in Table 1. 

After patients' admission to the ICU the glucose sensor was inserted into the adipose tissue in the abdominal region and continuous glucose monitoring was started after run-in period of 1h 45 min. The insulin infusion in both groups of patients started 1 h 45 minutes (sensor run-in period in eMPC-CGM group) after patients' arrival at the ICU from the operating theater and lasted for 24 hours. No routine protocol was used for perioperative glucose control. 

eMPC algorithm and continuous glucose measurement were implemented by the ICU nursing staff with supervision of an ICU physician as required. Protocol training was carried out by the ICU physician and a departmental nurse, usually individually, at bedside. 

### 2.2. Informed Consent

A written informed consent was signed by all participants before being enrolled into the study. The study was approved by the Human Ethical Review Committee, General University Hospital, Prague, Czech Republic, and was performed in accordance with the guidelines proposed in the Declaration of Helsinki.

### 2.3. Target Glucose Range

The target glucose range was set to 4.4 to 6.1 mmol/L, a level, which has been shown to reduce mortality and morbidity in cardiosurgical critically ill patients [3]. 

### 2.4. Patients' Examination

Clinical parameters and patients' clinical history data including age, sex, race, height, weight, BMI, history of diabetes and other chronic diseases, and type of surgery were collected prospectively.

### 2.5. Blood Glucose Monitoring, Insulin Treatment Regimens, and Nutrition

Blood glucose (BG) was monitored and insulin was administered according to each protocol rules/suggestions. Undiluted arterial blood for measurement of BG was drawn from an arterial line, inserted for routine monitoring procedures. Whole blood glucose was analyzed by a standard point-of-care testing device (ABL 700, Radiometer Medical, Copenhagen, Denmark). 

Insulin (Actrapid HM, Novo Nordisk, Baegsvard, Denmark) was given into a central venous line as a continuous infusion in both groups. A standard concentration of 50 IU of insulin in 50 mL of 0.9% NaCl was used. In all patients, infusion of 10% glucose solution was initiated upon admission to ICU with a glucose dose of 2.5 g/kg of ideal body weight (height in centimeters minus 100) per hour and lasted for 18 hours, when normal oral food intake was started. In ventilated patients, the glucose infusion lasted longer than the monitored 24 hour. 

Adverse events, medication, and nutrition were continuously monitored and documented. 

### 2.6. Continuous Glucose Monitoring, eMPC Algorithm, and Their Combination

A real-time continuous glucose monitoring system, Guardian REAL-Time CGMS (MiniMed Medtronic, Northridge, CA, USA), was used for continuous glucose measurement. A subcutaneous glucose sensor was inserted under the skin in the abdominal region immediately after arriving in the ICU. The monitoring started after a run-in period of 1 h 45 min. Glucose was measured every 5 minutes and displayed on the monitoring unit. The system was calibrated using arterial blood glucose concentrations measured by a standard point-of-care testing device (ABL 700, Radiometer Medical A/S, Copenhagen, Denmark). 

The enhanced model predictive (eMCP) algorithm used in this study was described in detail elsewhere [9, 14]. Glucose concentration, insulin dosage, and carbohydrate intake were the input variables for the eMPC and the output was the insulin infusion rate. The eMPC was implemented on a laptop computer. Control group was treated by this algorithm alone, while the variable sampling interval for the next blood glucose measurement calculated by the eMPC was respected. Arterial glucose was used as input for the eMPC. For a detailed description of the eMPC algorithm see the Appendix. The main computer interface of eMPC is also shown in Figure 3.

In the eMPC-CGM group data from CGM were entered manually into the eMPC every 15 minutes, while the variable sampling interval was not respected. Each hour glucose value from continuous glucose monitor was compared to reference arterial glucose using the Clarke error-grid analysis (C-EGA) [15] and when clinically unacceptable (zone C, D, or E of C-EGA) reference glycemia was used as input for the eMPC and to recalibrate the Guardian REAL-Time. When no additional calibration was needed, the sensor was calibrated every 12 hours as recommended by the manufacturer. In case of sensor failure (i.e., inability to calibrate) the study was interrupted and TGC was resumed using reference glycemia. 

### 2.7. Outcome Measures

The performance of Guardian REAL-Time CGMS was evaluated using Clarke Error-Grid Analysis (C-EGA), a standard tool for assessing accuracy of glucose meters [15]. The number of additional recalibrations of each sensor and the number of sensor failures were recorded.

Endpoints for effectiveness assessment of the TGC protocols were as follows: entire study average glycemia level; time to the target range of 4.4–6.1 mmol/L (80–110 mg/dL); average blood glucose level after reaching the target range; time within, above and below the target range throughout the whole study period and after reaching the target range; number of hypoglycemic episodes (≤2.9 mmol/L). The percentages of time in the specific ranges were calculated as number of hours in the selected range in each patient/24 ∗ 100.

### 2.8. Statistical Analysis

Statistical analysis was performed using SigmaStat software (Jandel Scientific, USA). The results are expressed as mean ± standard error of the mean (SEM). The TGC protocols were compared using Student's *t*-test or Mann-Whitney Rank Sum test as appropriate. Significance level was set at *P* = 0.05.

## 3. Results

Baseline characteristics of both study groups are listed in Table 1. The groups did not differ with respect to age, race, BMI, type of surgery, history of diabetes mellitus, and arterial hypertension. Baseline blood glucose was significantly higher in the eMPC-CGM group. 

**Figure 1:**
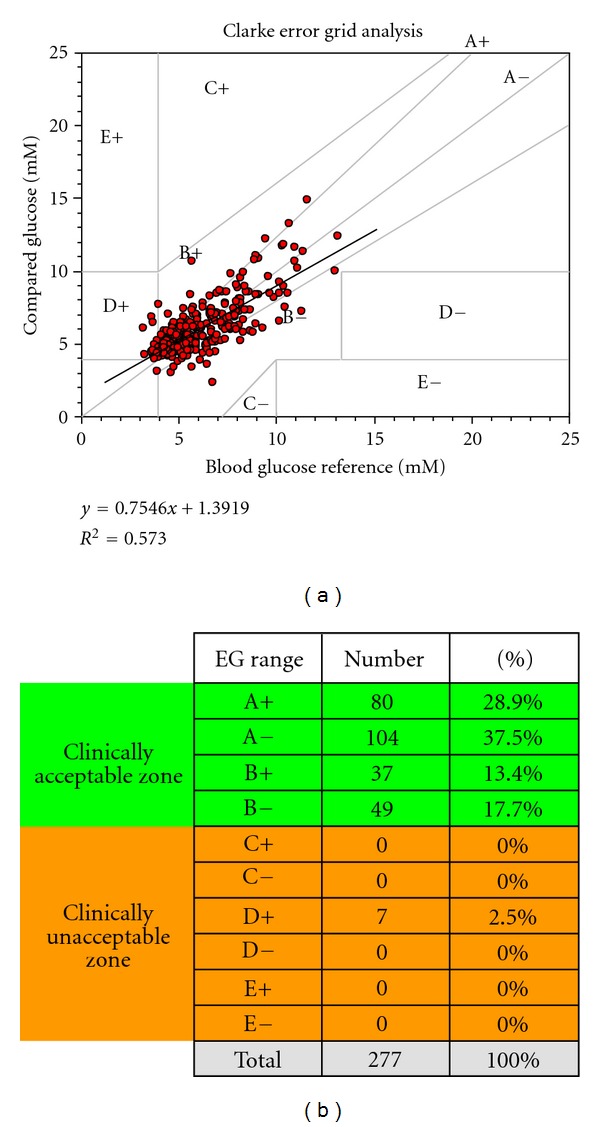
Clarke Error-Grid Analysis of data from Guardian RT during the whole study period. Zone A (accurate), within 20% of reference values, zone B (benign erroneous), outside of 20%, but not leading to inappropriate treatment, zones A and B, clinically acceptable accuracy. Zone C (unnecessary correction), leading to overcorrection of acceptable glucose levels, zone D, potentially dangerous failure to detect hypo- or hyperglycemia, zone E (erroneous treatment), erroneous treatment of hypo- or hyperglycemia (for hypoglycemia in case of hyperglycemia and vice versa), zones C+D+E—clinically unacceptable.

The performance of Guardian REAL-Time CGMS evaluated by C-EGA is shown in Figure 1. Of the 277 paired glucose values (values from the Guardian RT system and reference arterial glucose measured at the same time) obtained during the study, 270 (97.5%) were found in the acceptable A and B ranges of C-EGA (66.4% in A zone and 31.1% in B zone). Only 7 values (2.5%) were in the D zone with none of them being in the C and E zones. 

Of the 12 sensors used in the study (1 sensor for each patient), 6 needed no additional calibration except for the 2 obligatory ones (initially and after 12 hours). Of the other 6 sensors, 4 needed 1 extra recalibration, while the remaining 2 sensors had to be calibrated 3 or more times. One sensor failed after 21 hours, while the other 11 completed the designed 24-hour testing time.

**Figure 2:**
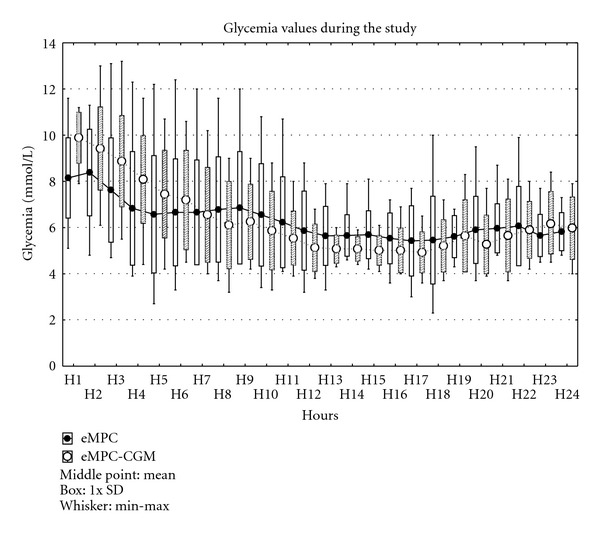
Glucose values in both groups throughout the study period. Values are means ± SD.

**Figure 3:**
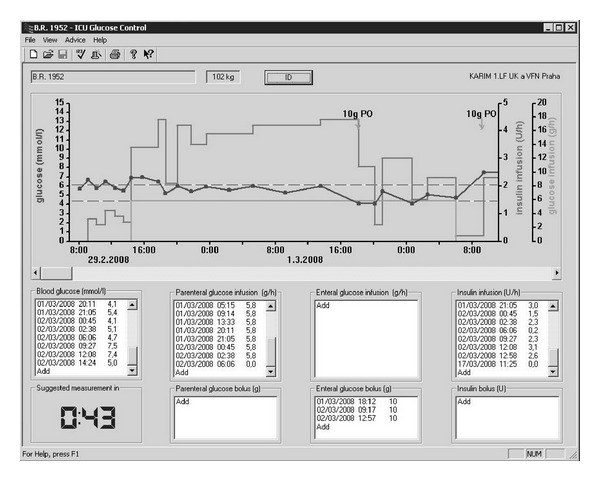


Performance of both TGC approaches using blood glucose-based endpoints is summarized in Table 2, while absolute glucose values throughout the whole testing period for both groups are depicted in Figure 2. The eMPC-CGM protocol showed similar glucose control compared to eMPC group as assessed by average blood glucose (6.2 ± 0.1  versus  6.1 ± 0.6 mmol/L, n.s.) and time spent in and above the target range throughout the whole study (46.3 ± 5.5 versus 46.2 ± 6.5 and 40.6 ± 5.9 versus 38.4 ± 5.1% of time, resp., n.s.) and also after reaching the target range. Time below the target range tended to be shorter in the eMPC-CGM group (13.1 ± 2.6 versus 15.4 ± 2.4 and 18.8 ± 3.8 versus 22.2 ± 4.6% of time, resp., n.s.), but without any statistical significance. Two episodes of severe hypoglycemia defined as blood glucose equal or below 2.9 mmol/L were observed in the control group, while no such episode was recorded in patients treated with eMPC-CGM protocol. Both hypoglycemic episodes were classified as “asymptomatic" and were not related to established major risk factors of ICU hypoglycemia such as nutritional interruption, asynchrony of nutrition and insulin administration, delayed glucose measurement, or drug administration. The combination of eMPC and Guardian REAL-Time tended to be more efficient in reaching the target levels of 4.4–6.1 mmol/L (7.6 ± 1.0 versus 8.8 ± 5.4 hours, n.s.).

## 4. Discussion

In the present study we tested the feasibility of a combination of an established computer-based protocol for tight glucose control (TGC) with a real-time continuous glucose monitoring system (CGM). This combination showed reasonable accuracy and reliability and resulted in similar glucose control as the computer-based algorithm alone. 

Compared with diabetic patients, where the precision of various CGM systems has been extensively tested, much less data is available for individuals with critical illness. [16–19]. Moreover, most of the studies that evaluated the performance of subcutaneous sensor-based CGM systems in ICU settings yielded conflicting results, with several trials reporting unsatisfactory correlation of continuous and systemic glucose [20, 21], insufficient accuracy of continuous systems [22], or underestimated hypoglycemia [23], whereas in others CGM systems provided clinically reliable measurements and correlated tightly with reference glucose values [24–29]. The accuracy of Guardian REAL-Time CGMS in our study with 97.5% values in the acceptable range of C-EGA was comparable to most of the data collected in other ICU trials and in routine diabetic patients. Nevertheless, it has to be stressed that subjects included into our study were specifically admitted for elective cardiac surgery. Therefore we cannot make general conclusions with respect to sensor performance in different, possibly more severely ill populations. Guardian REAL-Time CGMS sensors showed high reliability with 10 sensors requiring 0 to 1 calibration in addition to the standard 2 calibrations in 24 hours specified by the manufacturer for diabetic patients. Only 1 sensor failed to complete the whole 24-hour testing period. The sensors were well tolerated with no major local complications (significant bleeding, infection, irritation, pain). No serious technical or operational problems were recorded during the study.

The eMPC algorithm proved its effectiveness in maintaining target glycemia in several clinical trials [9, 30–32]. In a study recently conducted in our surgical ICU the performance of eMPC using intermittent glucose values was compared to two other TGC algorithms—the Matias protocol, which uses absolute glucose values, and the Bath algorithm based on relative glucose change. The eMPC protocol demonstrated the highest efficacy in achieving and maintaining glucose in the target range without excessive risk of severe hypoglycemic events [32]. In the present study the performance of the eMPC algorithm corresponded largely to results obtained in previous trials.

Only few studies have tried to combine CGM with TGM algorithms. A system using retrospective CGMS in a real-time manner coupled with a sliding scale algorithm in a closed-loop fashion developed by Chee et al. did not show significantly better performance compared to manual control [10]. In the so far largest trial evaluating real-time CGM in the ICU settings, including 124 mechanically ventilated patients and using a routine Leuven-derived protocol governed either by Guardian REAL-Time or intermittent arterial glycemia, CGM did not improve the allover glycemic control (time spent in target range, time to target range), although it significantly reduced number of hypoglycemic events [12]. 

In this study the eMPC-CGM combination resulted in similar glucose control compared to the use of eMPC algorithm alone as assessed by no significant differences in average glycemia and percentage of time in or above target range. The combined system required less time to reach the target levels and patients in the eMPC-CGM group tended to spend less time under the target range compared to the control group, but also without statistical significance. However, considering higher baseline blood glucose in the eMPC-CGM study arm, the inclusion of a CGM device seems to at least partially improve the performance of the eMPC algorithm. Moreover, no severe hypoglycemia (≤2.9) was observed in the eMPC-CGM group compared to 2 episodes in the eMPC group. These findings are of major importance in the light of recent large multicentric studies aiming at tight glucose control, which were discontinued due to excessive risk of hypoglycemia—the Glucontrol and the VISEP study [5, 7]—and particularly the NICE-SUGAR trial, where intensive insulin treatment targeted at normal glycemic levels was associated with an increased risk of hypoglycemia and overall mortality [6]. A large meta-analysis including all important TGC trials further confirmed a causal relationship between hypoglycemia prevalence and increased mortality. [33]. Therefore, our combination of eMPC and CGM seems to offer promising opportunities to achieve TGC goals in a safer manner, that is, without excessive risk of hypoglycemic episodes.

We are aware of several limitations of our study. As this was a study intended mainly at testing the practical feasibility of the proposed approach, the number of subjects in each study arm was relatively low. The potential of continuous glucose monitoring might not have been completely exploited, as continuous values were inserted into the eMPC in 15-minute intervals, even though they were updated every 5 minutes. Furthermore, the low rate of hypoglycemic events could be attributed to the relatively high constant rate of glucose infusion administered throughout the study. A constant high rate glucose infusion is expected to accelerate glucose turnover and the overall system response [34]. It is still possible that the overall outcome of the study would differ under the condition of a lower parenteral glucose administration and the results thus cannot be generalized. Finally, despite the absence of any severe hypoglycemic episode, the relatively long period of time spent under the target range in the eMPC-CGM group (in spite of being shorter than in the control group) might be of some concern as well.

## 5. Conclusion

In conclusion, the results of our pilot feasibility trial indicate that a combination of the computer-based enhanced model predictive control algorithm with continuous glucose monitoring by Guardian REAL-Time CGMS in cardiac surgery patients is reliable, accurate, and efficient enough to test this approach in larger populations. This treatment strategy might represent a further step towards a fully automated closed-loop system for insulin delivery in the critically ill, providing a temporary solution until the so-far largely experimental intravenous continuous glucose sensors are generally available.

## Figures and Tables

**Table 1:** Baseline characteristics of postcardiac surgery patients at the time of admission at ICU. Data are mean ± SEM.

	eMPC	eMPC-CGM
Number of patients (*n*)	12	12
Age (years)	67.5 ± 3.3	68.1 ± 2.2
Female (*n*)	4	6
Ethnicity: Caucasian (%)	100	100
BMI (kg/m^2^)	27.8 ± 1.0	29.1 ± 0.8
Type of surgery (*n*):		
CABG	6	8
Valve replacement	4	3
CABG + valve replacement	2	1
History of diabetes (*n*)	4	2
Previous insulin treatment (*n*)	2	0
Arterial hypertension (*n*)	11	11
Dislipidemia (*n*)	2	6

**Table 2:** The study blood glucose control data. Data are expressed as mean ± SEM. The percentages of time in the specific ranges were calculated as number of hours in the selected range in each patient/24 ∗ 100.

	eMPC	eMPC + Guardian	*P*
Baseline blood glucose	8.1 ± 0.6	9.9 ± 0.4	<0.05
The entire study blood glucose control data			
Average blood glucose (mmol/L)	6.1 ± 0.6	6.2 ± 0.1	n.s.
Time in target range (%)	46.2 ± 6.5	46.3 ± 5.5	n.s.
Time above target range (>6.1 mmol/L; %)	38.4 ± 5.1	40.6 ± 5.9	n.s.
Time below target range (<4.4 mmol/L; %)	15.4 ± 2.4	13.1 ± 2.6	n.s.
Severe hypoglycemia episodes (≤2.9 mmol/L)	2	0	
Blood glucose control data after reaching the target range (4.4–6.1 mmol/L)			
Average blood glucose (mmol/L)	5.2 ± 0.78	5.3 ± 0.1	n.s.
Time to target range (h)	8.8 ± 5.4	7.6 ± 1.0	n.s.
Time in target range (%)	62.8 ± 10.72	63.9 ± 5.4	n.s.
Time above target range (>6.1 mmol/L; %)	15.0 ± 8.6	17.3 ± 6.2	n.s.
Time below target range (<4.4 mmol/L; %)	22.2 ± 4.6	18.8 ± 3.8	n.s.
Severe hypoglycemia episodes (≤2.9 mmol/L)	2	0	

## Appendix

### The eMPC Algorithm

The eMPC includes a model of the glucoregulatory system, which adapts itself to the input-output relationship observed during tight glucose control; that is, an incoming glucose measurement is used by the model to update model parameters such as insulin sensitivity taking into account previously given insulin and parenteral and enteral glucose. Once individualized to a critically ill subject, the eMPC uses the glucoregulatory model to determine the optimum insulin infusion rate which is expected to achieve the target glucose concentration. This is achieved by numerical optimization using simulated experiments with the individualized glucoregulatory model. The output of this optimization is a sequence of insulin infusion rates which are expected, based on model predictions, to result in the target glucose concentration over a period of 4 hours. The first insulin infusion rate is displayed to the user and recommended for the delivery. The determination of the time-to-next glucose sample utilizes prediction accuracy. Through an internal procedure, the eMPC estimates how accurately it is able to predict glucose concentration. The extent of accuracy will differ over time as the unexplained variability in glucose concentration varies due to, for example, temporal variations in insulin sensitivity. The estimated prediction accuracy is used by the eMPC to plot a prediction envelope. This is a funnel-like prediction shape indicating a range of possible glucose concentrations at each time point in the future. Once the prediction funnel crosses a border indicating nonacceptable bounds, this might be a level indicating a risk of hypoglycaemia or unacceptable hyperglycaemia, the eMPC suggests a sample to be taken.

Glucose concentration, insulin dosage, and carbohydrate intake are the input variables for the eMPC. The insulin infusion rate and the time of the next glucose sample are the outputs. The eMPC was implemented on a bedside PC terminal.
